# Towards a Better Understanding of the Effects of UV on Atlantic Walruses, *Odobenus rosmarus rosmarus*: A Study Combining Histological Data with Local Ecological Knowledge

**DOI:** 10.1371/journal.pone.0152122

**Published:** 2016-04-06

**Authors:** Laura M. Martinez-Levasseur, Chris M. Furgal, Mike O. Hammill, Gary Burness

**Affiliations:** 1 Department of Biology, Trent University, Peterborough, Ontario, Canada; 2 Department of Indigenous Studies, Trent University, Peterborough, Ontario, Canada; 3 Environmental Resource Studies and Sciences, Trent University, Peterborough, Ontario, Canada; 4 Maurice Lamontagne Institute, Fisheries and Oceans Canada, Mont-Joli, Quebec, Canada; Smithsonian Institution, UNITED STATES

## Abstract

Walruses, *Odobenus rosmarus*, play a key role in the Arctic ecosystem, including northern Indigenous communities, which are reliant upon walruses for aspects of their diet and culture. However, walruses face varied environmental threats including rising sea-water temperatures and decreasing ice cover. An underappreciated threat may be the large amount of solar ultraviolet radiation (UV) that continues to reach the Arctic as a result of ozone loss. UV has been shown to negatively affect whales. Like whales, walrus skin is unprotected by fur, but in contrast, walruses spend long periods of time hauled-out on land. In this study, we combined the results of histological analyses of skin sections from five Atlantic walruses, *Odobenus rosmarus rosmarus*, collected in Nunavik (Northern Quebec, Canada) with qualitative data obtained through the interviews of 33 local walrus hunters and Inuit Elders. Histological analyses allowed us to explore UV-induced cellular lesions and interviews with experienced walrus hunters and Elders helped us to study the incidences and temporal changes of UV-induced gross lesions in walruses. At the microscopic scale, we detected a range of skin abnormalities consistent with UV damage. However, currently such UV effects do not seem to be widely observed at the whole-animal level (i.e., absence of skin blistering, erythema, eye cataract) by individuals interviewed. Although walruses may experience skin damage under normal everyday UV exposure, the long-term data from local walrus hunters and Inuit Elders did not report a relation between the increased sun radiation secondary to ozone loss and walrus health.

## Introduction

Walruses, *Odobenus rosmarus*, are one of the most emblematic species of the Arctic. The species has a discontinuous circumpolar Arctic and sub-Arctic distribution, and is represented by two subspecies: the Pacific walrus, *Odobenus rosmarus divergens*, which occurs in the Arctic and sub-Arctic waters of the Chukchi, Bering and Laptev seas (USA and Russia) [1,2], and the Atlantic walrus, *Odobenus rosmarus rosmarus*, which inhabits coastal areas from the north-eastern Canada, across Greenland (Denmark), Svalbard (Norway) and the western part of Arctic Russia (Barents and Kara seas) [3]. For thousands of years, walruses have been hunted by Northern Indigenous communities, which have relied on walruses and other marine mammal species for survival in the Arctic [4,5]. Today, walruses continue to be hunted by Northern communities, a right protected by agreements between federal and state governments and Indigenous peoples. Due to rapid environmental changes occurring in the Arctic, and due to the importance of walruses for Northern Indigenous communities [4,5], it is essential to survey and investigate walrus health, as well as identify emerging stressors that can affect walrus health.

Although ozone depleting substances have been controlled by the Montreal Protocol signed in 1987, polar ozone holes continue to form each year, and a large amount of solar ultraviolet radiation (UV) continues to reach our planet [6,7]. Additionally, the ozone layer above the Arctic is projected to be more sensitive to climate change than in the Antarctic [6]. In early 2011, for the first time in the observational record, the ozone hole over the Arctic was as large as the hole over the Antarctic [7]. While it is well known that unabsorbed solar ultraviolet radiation (UV) causes adverse effects in living organisms, [8–10], published studies on the effects of UV on wildlife remain limited to certain groups including amphibians [11], aquatic invertebrates, fishes [12–14] and more recently cetaceans [15–17]. For example, coral trout, *Plectropomus leopardus*, on the Australia Great Barrier Reef were observed with extensive skin cancer, likely due to the increased UV resulting from the Antarctic ozone depletion [12]. Meanwhile, recent work on cetaceans highlights the impacts of UV-induced damage on marine mammals [15,16]. Large whales, particularly those with light skin color (e.g., blue whales, *Balaenoptera musculus*), and those spending long periods of time at the sea-surface (e.g., sperm whales, *Physeter macrocephalus*), have been shown to develop skin lesions commonly associated with sunburn in humans [15]. Furthermore, whales with low melanin density, a photoprotective pigment produced by epidermal cells called melanocytes [18], were found to have higher levels of cellular lesions, suggesting that darker pigmentation confers cellular protection from sun irradiation in whales [15,16]. In contrast to most mammals, whales have evolved a smooth, imperfectly cornified epidermis uncovered by dense fur [19,20], which renders them sensitive to UV exposure. Whether other marine mammal species are similarly affected by UV exposure has not yet been explored.

Like whales, walrus skin is covered by a layer of sparse short hairs [2] and is not protected from the sun by thick fur, suggesting they may be vulnerable to extensive UV exposure. However, it is also possible that for equivalent UV exposure, the wrinkled and highly cornified skin of walruses [2] may be less sensitive to UV exposure than the skin of whales. Nonetheless, walruses spend long periods of time hauled-out on land and ice, for basking and breeding [2], likely resulting in UV exposure exceeding that of whales. Interestingly, Pacific walruses aggregating in summer coastal haul out sites tend to turn red [2,21]. Although it has been suggested that thermoregulation plays a role in the red color of these walruses aggregating on land [2,21], the effect may also be explained, at least in part, by excessive exposure to solar radiation, as observed in humans and laboratory animals exposed to UV for extended period of time [8,22,23]. In addition, if UV suppresses immunity in walruses, it could have consequences for walrus health, potentially resulting in increased rates of pathogen infection, as observed in humans and laboratory animals [24–26].

When working with species in remote locations such as the Arctic, where data collection is often challenged logistically and financially, there is increasing interest in combining scientific analyses with the documentation of traditional ecological knowledge (TEK) and/or local ecological knowledge (LEK; defined in methods) [27–32]. By using robust social research methods including interviews with local residents/experts [28,33–35], it is possible to produce a qualitative database providing valuable complementary information on aspects of wildlife ecology and physiology either overlooked or poorly understood by the scientific community [28–30,32]. For example, over the past 20 years, northern Indigenous peoples have reported changes in the intensity of the sun’s rays in the Arctic, and individuals have begun to report unusual sunburns and eye irritations [36]. Furthermore, Inuit Elders from Nunavik (Northern Quebec, Canada) have described increases in the incidence of sunburn in harbor seals, and women involved in tanning skins have reported the seal’s skin color becoming darker over the years [37].

Using histological analyses of skin samples collected from five Atlantic walruses hunted in Nunavik waters (Northern Quebec, Canada), our study aimed to better understand the effects of UV on walruses by addressing the following questions: Do walruses present cellular lesions similar to those previously reported in whales [15,16] and, if so, are these cellular lesions more abundant in those regions of the body that experience greater sun exposure? At the same time, we interviewed local walrus hunters and Elders to determine if they had observed gross skin lesions, skin redness, or eye cataracts, which might result from excessive or chronic UV exposure. And if so, whether any change in the frequency of these lesions has been noted in recent decades.

## Materials and Methods

To increase our understanding of the effects of UV on Atlantic walruses, we used a mixed methods design in which both quantitative and qualitative methods were used [34]. Analysis of each method was done separately and then brought together in a final integrated analysis [34].

### Walrus sampling and laboratory analyses

**Fig 1:**
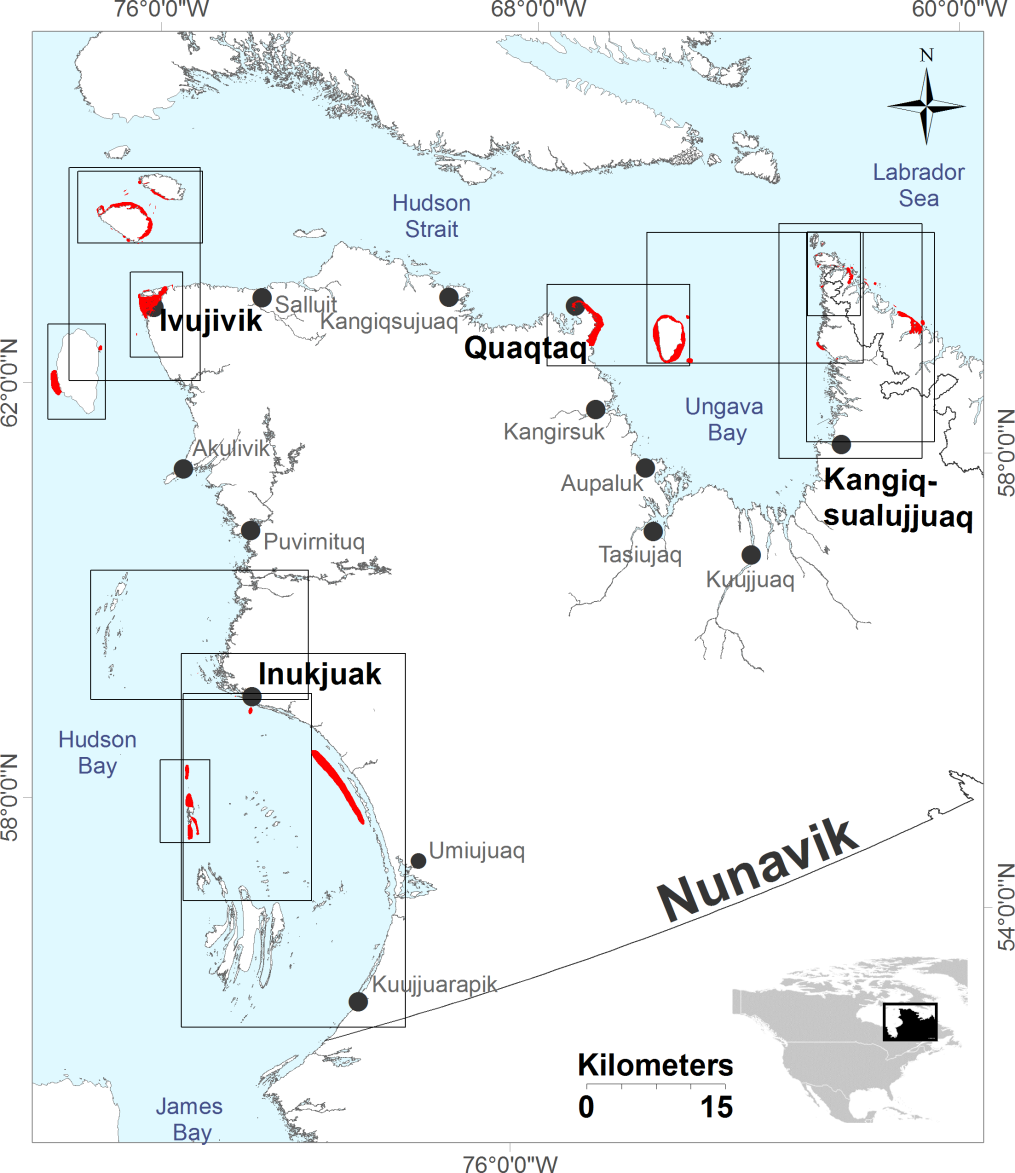
Map of Nunavik (Northern Quebec, Canada), showing the four communities involved in the project (Inukjuak, Ivujivik, Quaqtaq and Kangiqsualujjuaq). The quadrat show the limits of the base maps used to gather spatial data of walruses during the interviews (e.g., where walruses have been observed). Base maps in both English and Inuktitut of the areas surrounding participating communities were created using the geographic information system software ArcMap 10.1 (Digital vector datasets: RNCan-National Topographic Database). The scale of the maps, varied between 1:100,000 and 1:450,000 depending on the extent of walrus hunting areas provided by the local Hunters Fishers and Trappers Association during our first visit. A large scale, regional map (scale: 1:2,000,000) was also created. The mapping process of the interviews followed guidelines previously described [38]. The red areas correspond to the areas where walruses were reported by participants. The five walruses sampled in the study were hunted in the red area around Quaqtaq (latitude between 60.3975°N and 61.0775°N, longitude between 69.6339°W and 68.1703°W).

**Fig 2:**
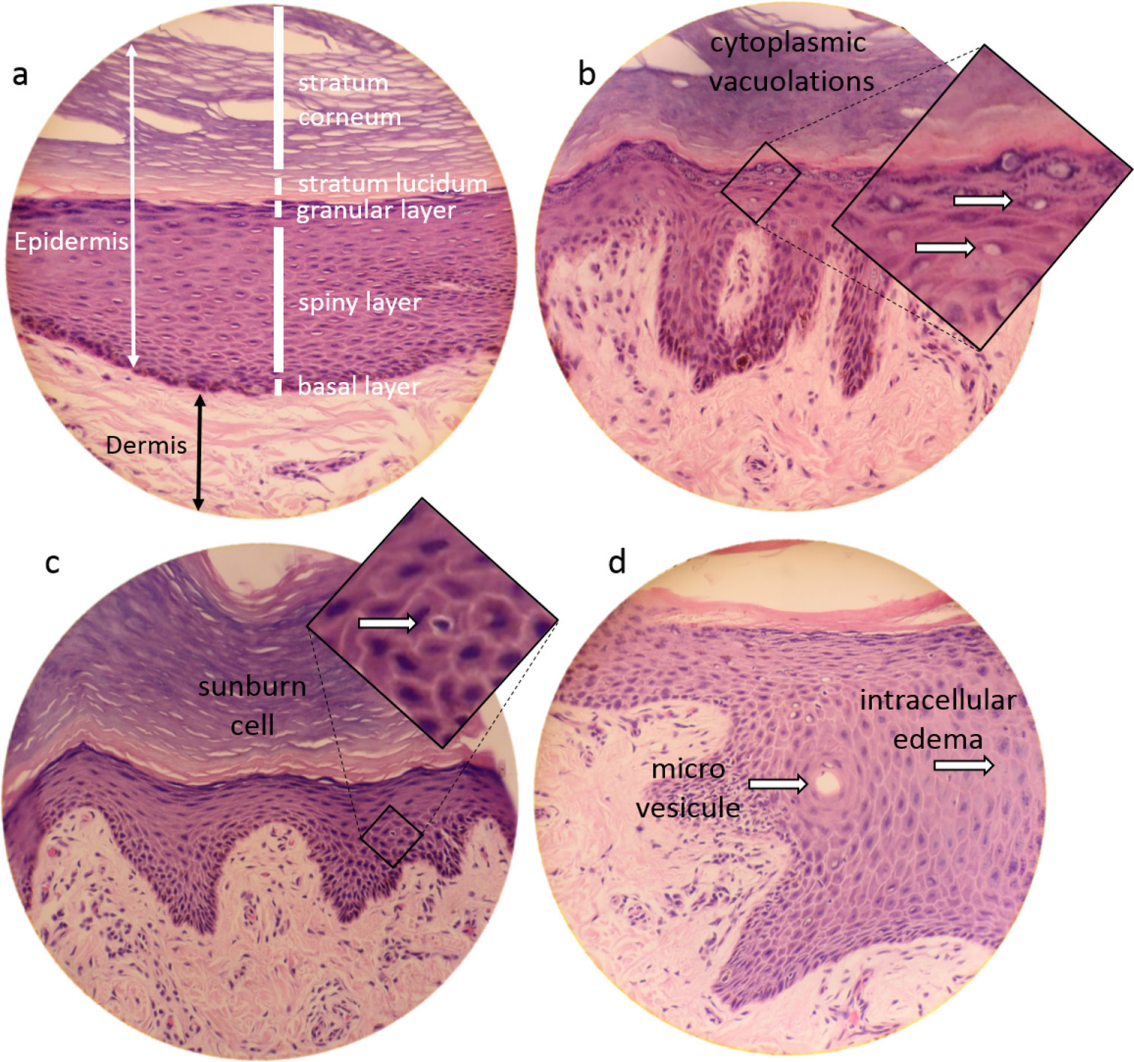
Sections of the dorsal skin of three walruses stained with H&E (microscope magnification X40; b & d correspond to the same walrus). a) The different layers of walrus’ skin. b) Examples of cells showing cytoplasmic vacuolation (indicated by arrows). c) An example of a sunburn cell (note the shrunken nucleus indicated by the arrow). d) An example of a microvesicle and intracellular edema (indicated by arrows). Walruses’ skin samples were collected during the Inuit subsistence walrus hunt near Quaqtaq in July 2013 and 2014.

In total, 10 skin samples were collected from five Atlantic walruses (three males and two females) in July 2013 (n = 4) and July 2014 (n = 1), as part of the Inuit subsistence hunt in Hudson Strait, near Quaqtaq, Nunavik (Fig 1). Two animals were sampled by LMML and three by local collaborators. One skin sample was collected from each of the ventral and dorsal regions, anterior to the transverse plane, of each walrus. The ventral region was designed to act as a negative control, as this region is presumably exposed to little direct UV (other than reflectance). Each sample (cube of 1cm^3^) included the epidermis and dermis (Fig 2A). Upon collection, samples were preserved in the field in 10% buffered formaldehyde solution for later histological analyses. Of the 10 samples collected, one sample was excluded from the analyses, because the sampling location on the body was uncertain (see raw data in S1 Table). Although data on sex and age category (four levels: pup, juvenile, adult, old individual) were provided for each walrus, due to the relatively low number of individuals (n = 5), age categories and sexes were pooled. Samples were transported to Trent University under permits issued by The Department of Fisheries and Oceans, Canada.

Skin sections were prepared by the Animal Health Laboratory of the University of Guelph. Briefly, the skin fixed in formalin was dehydrated through a series of alcohols, followed by xylene, before being embedded in paraffin wax. The blocks of skin embedded in wax were then sectioned at 4–5 μm and stained with Haematoxylin and Eosin (H&E). Slides were analyzed under 40X magnification, and lesions were semi-quantified as previously described [15]. Binary response categories (Presence/Absence: 0 = absence and 1 = presence; Levels: 0 = absent or low and 1 = high and widely distributed) were created for each lesion, as well as for the photoprotective pigment melanin.

### Documentation of local ecological knowledge (LEK)

Although local ecological knowledge and understanding of the environment by Indigenous peoples is often referred to in the literature as traditional ecological knowledge, or TEK [27–29,39], we concur with previous authors [40–42] that TEK may not be the most appropriate to describe the information presented in studies that report solely contemporary observational data of participants acquired over a lifetime rather than knowledge and understanding transmitted over generations. Furthermore, detailed ecological information of local environments has been reported to be held by other non-Indigenous groups elsewhere around the world [43–45]. As we are reporting observations of participants during their lifetime and include one non-Inuk hunter recognized as a walrus expert by the local Hunters Fishers and Trappers Association, we use the term local ecological knowledge (LEK), as used elsewhere in the literature [40,44], to describe the knowledge documented in our study.

Between June and September 2013 we interviewed 33 walrus experts recognized for their knowledge and activity in walrus hunts by their communities and the local Hunters Fishers and Trappers Associations of Inukjuak, Ivujivik, Quaqtaq and Kangiqsualujjuaq in Nunavik (Northern Quebec, Canada) (Fig 1). Semi-directed interviews [28,33] were conducted with the help of English-Inuktitut interpreters. In each community, we interviewed between 7 and 10 participants, ranging from 35 to 85 years of age. The age of the participants was distributed in the following age groups: 35–55, 56–65, 66–75 and 76–85 years old (respectively 7, 7, 12 and 7 participants). Elders older than 75 years of age shared knowledge and observations that extended back to the 1930s, and thus represented eight decades of experience and observations. Because women are involved in walrus meat preparation and thus spend more time looking at the organs including the skin of the animals, four women were included among the 33 interviewees. Although we recognize that women and men can hold different kinds of knowledge [46], the sample size for women was small and the information provided by women and men was qualitatively similar. As such, we did not separate these data by gender in our analyses.

Semi-directive interviews were used to document observed changes in walrus health, including skin lesions, eye cataracts and changes in skin color that might result from increased sun exposure. In order to increase precision in knowledge exchange between experts and interviewers, a guide showing photographs of eye cataracts, different levels of skin lesions, and skin colors was created (copies of the guides are available from corresponding author). For example, we used a 5-point scale of walrus skin color, from light brown to red (specifically light brown, dark brown, black, light pink, red) and asked participants which skin colors they had seen. Because we were interested in whether skin lesions similar to the ulcerative lesions of unknown etiology reported in Pacific walruses, *Odobenus rosmarus divergens*, in Alaska in 2011 [47] had been observed in Atlantic walruses while in Nunavik waters, we used previously published photographs of the lesions during the interviews (S1 Fig). The audio-recorded interviews were transcribed and the text was then entered into the qualitative analytical software program NVivo (Version 10, 2012; QSR International) and analyzed using thematic content analysis [34]. To ensure the information provided by participants had been interpreted accurately, data were verified and validated during subsequent workshops held with participants in July 2014. Final results and interpretation were then presented to each community in March 2015. As not all participants answered every question, sample sizes reported in the results section can be less than the total number of participants interviewed for the overall study.

The methods used in our study, including the selection of participants, the development of the consent form, questionnaire and interview support guides (all available from corresponding author), as well as the interviews and group validation workshops, followed the standards of social research methods used to document local knowledge [28,33–35]. This project was approved by the four Inuit communities & their Local Hunting Fishing & Trapping Associations, Northern Villages & Landholding Corporations (March-September 2013), the Nunavik Marine Region Wildlife Board (December 2012) and by Trent University Research Ethics Board (December 2012) and the Trent Aboriginal Education Council (February 2013). All participants have provided written informed consent.

## Results

### UV-induced cellular lesions (laboratory analyses)

**Fig 3:**
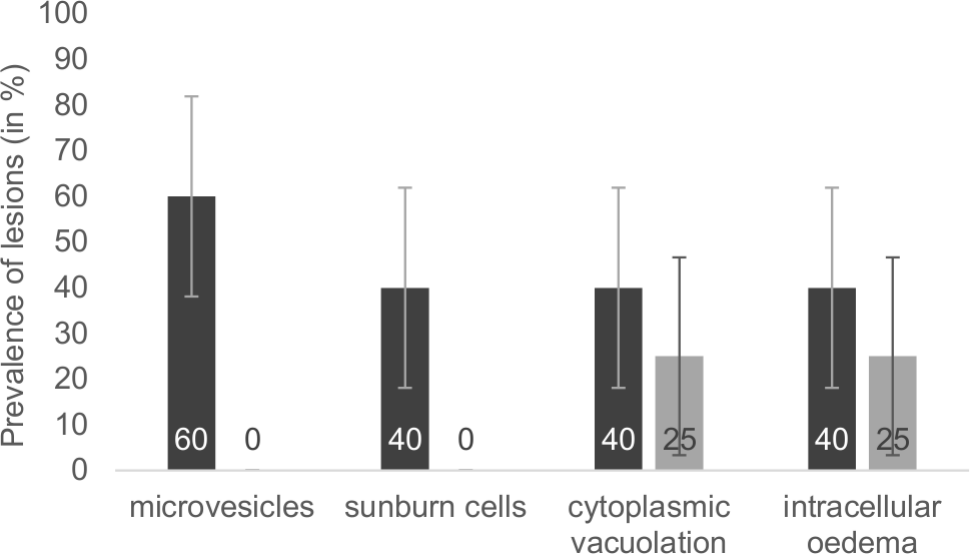
Presence of cellular lesions observed in the samples obtained from the dorsal region of the walruses (dark grey bars; total number of dorsal samples = 5) and the samples obtained from the ventral region of the walruses (light grey bars; total number of ventral samples = 4). The binary response data used for microvesicles were: zero = absence; one = presence, and for sunburn cells, cytoplasmic vacuolation and intracellular edema: zero = absent or low levels, and one = high levels and widely distributed. Raw data are available in S1 Table. Percentages are provided on the bars. Bars ± SE.

Histological analysis of walrus skin sections revealed a range of abnormalities, including microvesicles, intracellular edema, cytoplasmic vacuolation and sunburn cells (Fig 2B–2D). Interestingly, while 60% (3 of 5) and 40% (2 of 5) of the dorsal skin sections presented microvesicles and high levels of sunburn cells, none or low levels were detected in skin sections from the ventral region (Fig 3; raw data available in S1 Table). A similar trend was observed for cytoplasmic vacuolation and intracellular edema (Fig 3; raw data available in S1 Table). No leukocyte infiltration was observed.

### Skin pigmentation (laboratory analyses & LEK)

#### Melanin (laboratory analyses)

**Fig 4:**
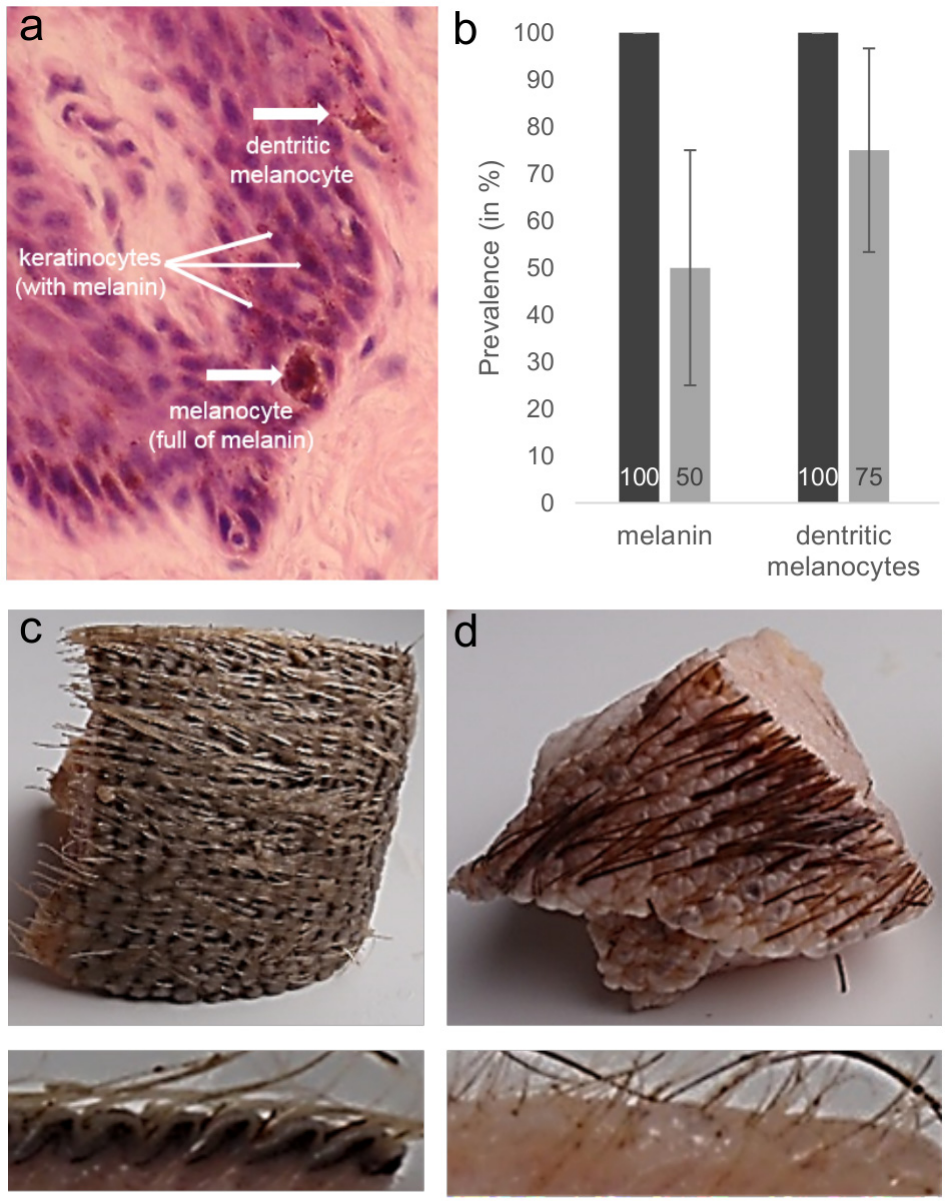
Walrus skin pigmentation. a) Walrus’ dorsal skin sections showing the presence of melanin in the epidermis. Melanin is produced in the melanocytes, which become dendritic to distribute the melanin to the neighbor epidermis cells called keratinocytes. b) Prevalence of melanin and dendritic melanocytes observed in the samples obtained from the dorsal region of the walruses (dark grey bars; total number of dorsal samples = 5) and ventral samples (light grey bars; total number of ventral samples = 4). The binary response data used for melanin and dendritic melanocytes were: zero = absence; one = presence. Prevalence is provided on the bars. Bars +/- SE. Raw data are available in S1 Table. c) and d) Skin samples of around 1cm^3^ obtained from the dorsal (c) and ventral (d) regions of the walrus body. The photographs show the highly pigmented skin (dark color) of the dorsal sample compared with the less pigmented skin of the ventral sample (light color).

While all five dorsal skin sections contained the photoprotective pigment melanin, only 50% of the ventral skin sections had melanin (2 of 4; Fig 4A and 4B; raw data available in S1 Table). This is consistent with the observation that the pigmentation of the sample collected on the walrus’ dorsal region was darker than the sample collected from its ventral region (Fig 4C and 4D). Basal dendritic melanocytes were observed in all dorsal skin samples (5 of 5), but in only 75% of the ventral skin sections (3 of 4; Fig 4A; raw data available in S1 Table).

#### General body color (LEK)

The majority of participants (97%; 31 of 32) reported that the walruses they observed in Nunavik were mostly black and dark brown. Only 41% of the 32 participants reported having seen light pink walruses. Participants added they were relatively rare. Similarly, light brown walruses were also infrequently reported by participants. Finally, no participants (0 of 32) reported having seen red walruses. Interestingly, 86% of participants (19 of 22) reported that walrus skin color changes with age, with young walruses being darker than old walruses. Light pink walruses were reported by 18% of participants (4 of 22) to be most likely old male walruses.

### Gross skin lesions and eye cataracts (LEK)

#### Gross skin lesions

Seventy percent of participants (21 of 30) reported having observed skin lesions on walruses (specifically the lesions shown in S1B Fig). Only 7% of participants (2 of 30) reported having observed the lesions depicted in S1A Fig. Finally, one participant reported observing the lesions shown in S1E Fig, located all over the body of an abnormally lean walrus found lying alive on a rock.

#### Eyes cataracts

Cataracts in the eyes of walruses were reported by only 6% of participants (2 of 33), who explained that cataracts were mostly observed in older individuals. On the contrary, 76% of participants (25 of 33) reported walruses with red eyes. Only 15% of participants (5 of 33) said that walruses’ eyes were not always checked after a kill by hunters.

### Temporal changes (LEK)

#### Absence of temporal changes in walrus skin color and lesions

No participant (0 of 32) reported a temporal change in color or number of skin lesions or eye cataracts in the walruses they have observed over their time hunting.

#### Evidence of increased sun exposure in Nunavik

In general, participants agreed that the intensity of the sun had increased in their region. No participants (0 of 32) reported having observed sunburn in walruses, but 27% of participants (8 of 30) reported observing seals with sunburn. Lucassie Kanuarjuak, an Elder from Ivujivik explained that *“when seals are basking in the sun during the spring time*, *the sun affects the fur*, *the skin*. *[…] The seals are burnt by the sun*. *I know that for fact*.*”* Quitsaq Tarriasuk, an Elder from Ivujivik, stated that *“the seals do get sunburned*. *[…] It becomes an unsightly appearance on its skin*, *losing its hair and the coloration is somewhat affected too*, *it looks very different from a healthy seal*. *[…] Obviously those are spending more time basking in the sun […] during the months of May and June*.*”* Unfortunately, these two participants were absent from the discussion held during the validation workshop, about the difference between seal sunburn and seal molting. Thus, we could not confirm whether these observations were simply a description of the annual seal molting process. Actually, during the validation workshop, Johnny Oovaut from Quaqtaq explained that one of the names for seal in Inuktitut, *uutuq*, means “*something getting burnt*.”

## Discussion

Due to the unprecedented ozone loss recorded in the Arctic in 2011 [7], it is of critical importance to understand the extent of damage caused by natural UV exposure in Arctic species, particularly those of economic or cultural significance such as walruses [4]. However, such an objective is challenging mainly because of the difficulty in obtaining samples. For this reason, we combined the results of histological analyses of skin sections from five Atlantic walruses with qualitative data obtained through the interviews of 33 local walrus hunters, including women and Inuit Elders.

The number of skin samples we collected was relatively small, but we nonetheless detected a range of skin abnormalities, including microvesicles, intracellular edema, cytoplasmic vacuolation and sunburn cells, as previously reported in whales [15]. These abnormalities are generally observed 24 hours after sun-exposure in humans and laboratory animals [22,48,49], suggesting that the lesions we observed might result from sun exposure. While similar cellular lesions were negatively correlated to levels of pigmentation in whales, providing further evidence of their sunburn characteristics [15,16], in the current study such a relation could not be tested for due to the low number of samples. However, as predicted, skin samples collected from dorsal region of the walruses, corresponding to the area of the body most exposed to the sun, exhibited higher levels of lesions than samples collected from the ventral region. Interestingly, higher levels of melanin, a photoprotective pigment produced by melanocytes [18] and which likely provides sun protection, were observed in dorsal skin samples. In addition, basal dendritic melanocytes were observed in all dorsal skin samples, but in only 75% of the ventral skin sections, suggesting that melanocytes are more active in sun exposed-areas. Indeed, melanocytes generally become dendritic (with branched projections) when producing and distributing the photo-protective melanin pigments to the rest of the epidermis [18].

When over-exposed to UV, humans and laboratory animals develop erythema, corresponding to the redness of the superficial skin resulting from dermal vasodilatation [22,23]. Pacific walruses aggregating in summer coastal haul-outs tend to turn red, which has been attributed to the vasodilatation of the superficial capillaries to release excessive heat [2,21]. Although thermoregulation likely plays a role in the red color of the Pacific walruses aggregating on land, we hypothesized that solar ultraviolet radiation may partly explain the apparent erythema observed. In contrast to our hypothesis, observations of light pink Atlantic walruses were relatively rare, and no red Atlantic walruses were reported by the walrus hunters and Elders we interviewed. It is possible that the low number of reported light pink walruses, and the absence of red walruses, is because hunters typically pursue swimming walruses, and thus spend less time observing basking walruses. However, the absence of reported red walruses in Nunavik (0 observations among 32 participants, some of whom had knowledge and observations dating back to the 1930s) suggests that red walruses are only observed in other regions (e.g., Pacific region) [21]. It would be interesting to investigate whether there exists variation in walrus skin coloration among regions, particularly between Atlantic and Pacific walruses, and whether such variation corresponds with different levels of sunburn sensitivity, as has been observed in humans [23,50]. However, such a study would need to control for potential regional variability in UV levels.

Participants reported that the skin color of the walruses they observed changes with age, with young walruses being darker than old walruses. This suggests that Atlantic walruses also become lighter with age, as previously reported for Pacific walruses [2]. If walruses lose skin pigmentation with age, then older individuals might be more sensitive to solar radiation, as observed in humans [51]. This could partly explain why 18% of participants said that light pink walruses were generally old males. Although, it is possible that walruses can develop sunburn lesions such as blistering [22], those might be difficult to discern due to the highly cornified and wrinkled skin of walruses [2]. Furthermore, walrus skin is constantly injured by fights amongst walruses or predators [2]. For these reasons, skin lesions not resulting from fights or skin diseases might be under-diagnosed.

In 2011, ulcerative skin lesions of unknown etiology were reported in 74% of Pacific walrus carcasses (14 of 19 individuals) found at an annual haul-out site at Point Lay, Alaska, USA [47]. The lesions were round-to-irregularly shaped, and were distributed over the head, trunk and limbs of the affected animals. While some lesions were “weeping blood”, suggesting the early stages of wound development, others were hypopigmented scars indicating ending stages of healing [47]. Because the walrus skin lesions had a similar appearance to the lesions observed on the unusual number of stranded seals (over 100, mainly ringed seals, *Pusa hispida*) in the Arctic and Bering Strait regions of Alaska for the same year, walruses were included in the Pinniped Unusual Mortality Event (UME). Although rigorous investigation was performed by the UME working group [52,53], the cause of the lesions remains unclear. While new cases have been reported for Alaskan seals, no new walrus cases have been reported, suggesting that any unusual environmental conditions that could have contributed to the event are no longer present [54]. When shown images of the lesions on Pacific walruses, Inuit participants reported observing similar skin lesions in Atlantic walruses, but explained that in Atlantic walruses the lesions were mainly the result of tusk wounds due to fighting. From the interviews, it was difficult to know whether the lesions reported by participants were the same as those observed during the Unknown Mortality Event (UME) that occurred in 2011 in Alaska [47]. However, it was clear that Inuit participants did not believe that the lesions they had observed were the result of intensive sun exposure.

Eye cataracts were rarely observed in walruses, and when observed, participants reported they were found mostly in older individuals. Previously, it has been shown that captive pinnipeds have a higher risks of developing cataracts [55] when they are older than 15 years of age, have a history of fighting or ocular disease, and/or insufficient access to UV-protective shade. Because Inuit hunters try to select healthy looking animals, and usually avoid walruses with damaged skin or old individuals, it is probable that the rate of cataract occurrence we report is an under-representation of that which might be present in Atlantic walruses around Nunavik. It is also possible that the eyes of walruses are not always checked by hunters, as explained by 15% of participants. However, some participants disagreed and explained that if there were something abnormal in the eyes of a killed walrus, it would be noticed. In fact, 76% of participants reported walruses with red eyes, suggesting that hunters do notice ocular characteristics. As a result, it seems likely that if there were a high proportion walruses with advanced cataracts, hunters would have detected and been able to report them.

Finally, although no participants reported a temporal change in skin color, or the number of skin lesions or eye cataracts in the walruses that have been observed since the 1930s, they agreed that the solar radiation intensity has increased. This corroborates results of community workshops held across the Canadian Arctic, including Nunavik, Nunavut, Inuvialuit Settlement Region and Nunatsiavut, in which it has been reported that northern residents have increasingly experienced unusual sunburn and eye irritation beginning in the early 1990s [36,37]. Additionally, scientists working in the Canadian high-Arctic who monitored their personal UV-exposure using dosimeters [56], were found to have been exposed to UV levels high enough to result in a significantly increased risk of skin cancers, such as melanoma [9]. Northern residents have also reported changes in wildlife skin, including that of caribou, *Rangifer tarandus*, and harbor seals, *Phoca vitulina*, likely as a result of increased sun intensity. For example, hunters have noticed a change in the texture of the dorsal skin of caribou, specifically around the neck [36]. Inuit Elders from Nunavik (Northern Quebec, Canada) have described increases in the incidence of sunburn in harbor seals, and women involved in tanning skins have reported the seal’s skin color becoming darker over years [37]. These observations highlight the changes occurring in the Arctic, likely resulting from increased solar radiation. Although concerns about the effects of UV on Arctic wildlife are increasing, published papers remain scarce (but see [57], for a review of the potential effects of UV on Arctic freshwater and anadromous fisheries). Our study, bringing together both histological analyses and local ecological knowledge of walrus hunters and Elders, is the first to attempt to study the effect of solar exposure on Arctic mammals, and we hope it will stimulate further investigation in this emerging research field. For example, it would be interesting to compare levels of UV-induced cellular lesions in Atlantic walrus skin throughout the season, particularly at the beginning of their spring migration, and after their migration when resting at their summer coastal haul-outs. Indeed, walruses basking for many hours at coastal haul-outs in August might be more severely affected by the sun than the walruses migrating in July, which is when samples were obtained in this study.

## Conclusions

Although, the number of walrus skin samples collected was relatively small, we detected at the microscopic scale a range of skin abnormalities consistent with UV damage. However, such UV effects do not seem to be widely observed, at least by local observers, at the whole-animal level (i.e., absence of advanced erythema, skin blistering or eye cataracts). It is possible that the wrinkled and highly cornified skin of walruses plays a role in UV-protection, and thus protects walrus skin from blistering. It is also possible that gross lesions were under-diagnosed in walruses. Finally, while local observers agreed that the intensity of sun radiation has increased, they did not report any temporal change in the general appearance of walruses that have been observed since the 1930s (e.g., darkening of walrus skin color). Although walruses may be getting burned under normal everyday UV exposure, the long-term data from local hunters and Elders did not show a decrease in the condition of the skin of the Atlantic walruses that could have been linked with the increased sun radiation secondary to ozone loss. Finally, in an integrated analysis such as this, it is important to recognize that the different knowledges presented are rooted in different epistemologies and often have different foci of interest [58]. These fundamental differences can also provide an explanation for potentially contradicting evidence or unique contributions from each knowledge system.

## Supporting Information

S1 Fig Photographs of the skin ulcerative lesions of unknown aetiology reported in Pacific walruses, *Odobenus rosmarus divergens*, and used during the interviews with local hunters from Nunavik (Quebec, Canadian Arctic) to find out whether they observe similar lesions in the Atlantic walruses, *Odobenus rosmarus rosmarus*, they hunt for subsistence. Lesions were observed at the Point Lay, Alaska, 2011 and in Chukotka, Russia, 2009. Photo credits: A and B Fischbach (U.S. Geological Survey, Alaska Science Center Walrus Research Program), D J Garlich Miller (U.S. Fish and Wildlife Service, Marine Mammals Management), C A Kochnev (TINRO center; current place of work: Beringia National Park, Institute of Biological Problems of the North Far East Branch, Russian Academy of Sciences), E R Stimmelmayr (North Slope Borough—Department of Wildlife Management). Permissions have been granted by the authors to use their photos in this paper. This figure has been modified from: Garlich-Miller J, Neakok W, Stimmelmayr R. Field Report: Walrus Carcass Survey, Point Lay Alaska, September 11–15, 2011. 2011. Available:http://www.fws.gov/alaska/fisheries/mmm/walrus/pdf/2011_point_lay_walrus_carcass_survey.pdf.
(PDF)

S1 Table Raw data for the presence and levels of skin abnormalities detected at the microscopic scale in the ventral and dorsal regions of five Atlantic walruses, *Odobenus rosmarus rosmarus*. In total, 10 skin samples were collected from five Atlantic walruses (three males and two females) in July 2013 (n = 4) and July 2014 (n = 1), as part of the Inuit subsistence hunt in Hudson Strait, near Quaqtaq (Nunavik, Northern Quebec, Canada). Two animals were sampled by LML and three by local collaborators. One skin sample was collected from each of the ventral and dorsal regions of each walrus. The ventral region was designed to act as a negative control, as this region is presumably exposed to little direct UV (other than reflectance). Each sample (cube of 1cm^3^) included the epidermis and dermis. Upon collection, samples were preserved in the field in 10% buffered formaldehyde solution for later histological analyses. Of the 10 samples collected, one sample (W3NU-S1) was excluded from the analyses, because the sampling location on the body was uncertain. Skin sections were prepared by the Animal Health Laboratory of the University of Guelph. Briefly, the skin fixed in formalin was dehydrated through a series of alcohols, followed by xylene, before being embedded in paraffin wax. The blocks of skin embedded in wax were then sectioned at 4–5 μm and stained with Haematoxylin and Eosin (H&E). Slides were analyzed by LML under 40X magnification, and lesions were semi-quantified following Martinez-Levasseur et al. (2011). Binary response categories (Presence/Absence: 0 = absence and 1 = presence; Levels: 0 = absent or low and 1 = high and widely distributed) were created. NA = not available.
(PDF)
