# Supplementary material for: Towards a Better Understanding of the Effects of UV on Atlantic Walruses, Odobenus rosmarus rosmarus: A Study Combining Histological Data with Local Ecological Knowledge
Source: PLoS One. 2016 Apr 6;11(4):e0152122. doi: 10.1371/journal.pone.0152122 (PMC4822789; doi:10.1371/journal.pone.0152122)
Supplement: S1 Fig — Lesions were observed at the Point Lay, Alaska, 2011 and in Chukotka, Russia, 2009. Photo credits: A and B Fischbach (U.S. Geological Survey, Alaska Science Center Walrus Research Program), D J Garlich Miller (U.S. Fish and Wildlife Service, Marine Mammals Management), C A Kochnev (TINRO center; current place of work: Beringia National Park, Institute of Biological Problems of the North Far East Branch, Russian Academy of Sciences), E R Stimmelmayr (North Slope Borough—Department of Wildlife Management). Permissions have been granted by the authors to use their photos in this paper. This figure has been modified from: Garlich-Miller J, Neakok W, Stimmelmayr R. Field Report: Walrus Carcass Survey, Point Lay Alaska, September 11–15, 2011. 2011. Available:http://www.fws.gov/alaska/fisheries/mmm/walrus/pdf/2011_point_lay_walrus_carcass_survey.pdf. (PDF) [file pone.0152122.s001.pdf]

SUPPORTING INFORMATION

File content: The supporting information contains one figure entitled: Photographs of the skin ulcerative lesions of unknown aetiology reported in Pacific walruses, *Odobenus rosmarus divergens*, and used during the interviews with local hunters from Nunavik (Quebec, Canadian Arctic) to find out whether they observe similar lesions in the Atlantic walruses, *Odobenus rosmarus rosmarus*, they hunt for subsistence.

Towards a Better Understanding of the Effects of UV on Atlantic Walruses, *Odobenus rosmarus rosmarus*: a Study Combining Histological Data with Local Ecological Knowledge

Laura M. Martinez-Levasseur[1,2,*], Chris M. Furgal[2], Mike O. Hammill[3], Gary Burness[1,*]

[1] Department of Biology, Trent University, Peterborough, Ontario, Canada
[2] Departments of Indigenous Studies and Environmental Resource Studies and Sciences, Trent University, Peterborough, Ontario, Canada
[3] Maurice Lamontagne Institute, Fisheries and Oceans Canada, Mont-Joli, Quebec, Canada

*Corresponding authors
E-mails: garyburness@trentu.ca (GB), lmmartinezlevasseur@gmail.com (LMML)

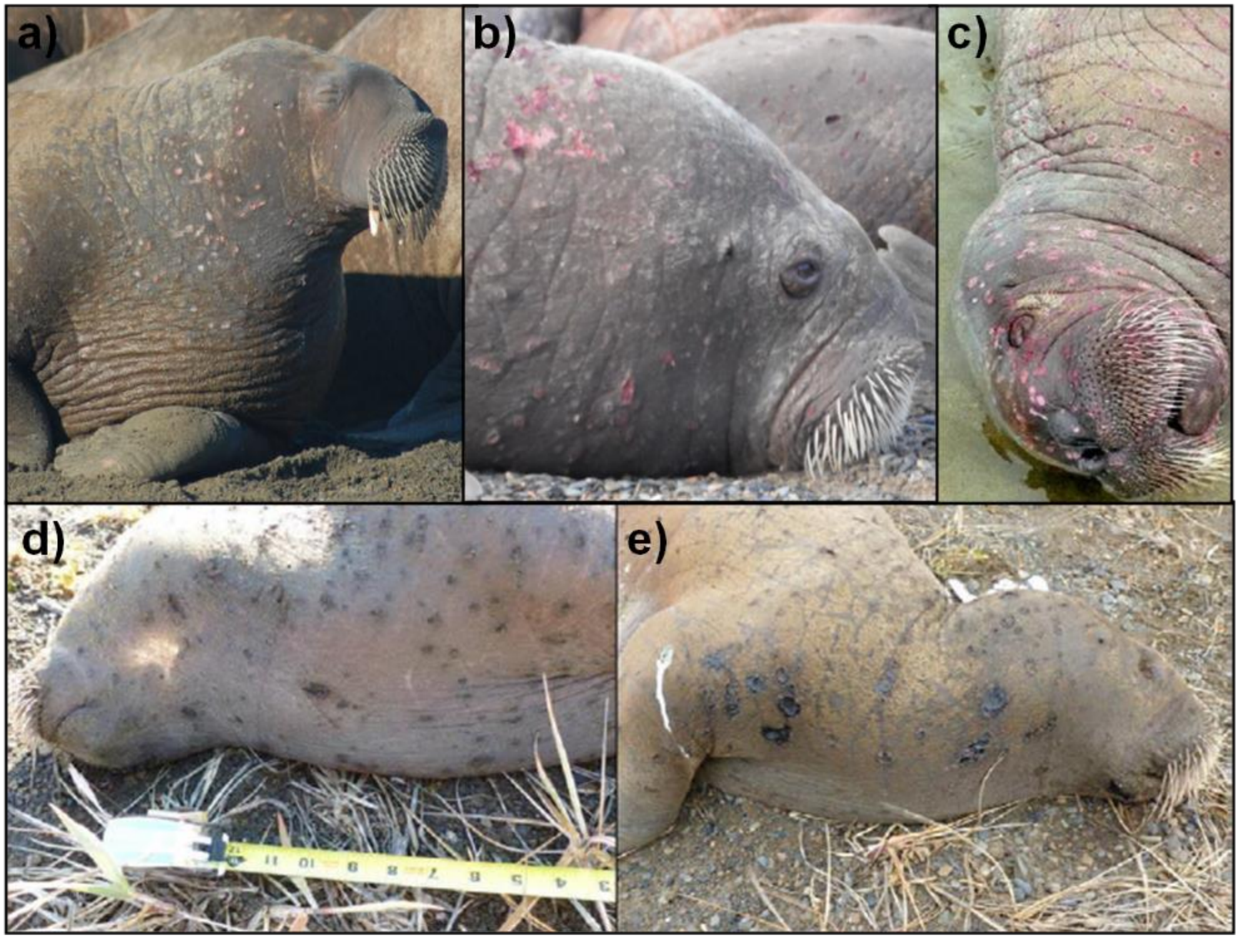

S1 Fig. Photographs of the skin ulcerative lesions of unknown aetiology reported in Pacific walruses, *Odobenus rosmarus divergens*, and used during the interviews with local hunters from Nunavik (Quebec, Canadian Arctic) to find out whether they observe similar lesions in the Atlantic walruses, *Odobenus rosmarus rosmarus*, they hunt for subsistence. Lesions were observed at the Point Lay, Alaska, 2011 and in Chukotka, Russia, 2009. Photo credits: A and B Fischbach (U.S. Geological Survey, Alaska Science Center Walrus Research Program), D J Garlich Miller (U.S. Fish and Wildlife Service, Marine Mammals Management), C A Kochnev (TINRO center; current place of work: Beringia National Park, Institute of Biological Problems of the North Far East Branch, Russian Academy of Sciences), E R Stimmelmayr (North Slope Borough - Department of Wildlife Management). Permissions have been granted by the authors to use their photos in this paper. This figure has been modified from: Garlich-Miller J, Neakok W, Stimmelmayr R. Field Report: Walrus Carcass Survey, Point Lay Alaska, September 11-15, 2011 [Internet]. 2011. Available:http://www.fws.gov/alaska/fisheries/mmm/walrus/pdf/2011_point_lay_walrus_carcass_survey.pdf.
