# Supplementary material for: Towards a Better Understanding of the Effects of UV on Atlantic Walruses, Odobenus rosmarus rosmarus: A Study Combining Histological Data with Local Ecological Knowledge
Source: PLoS One. 2016 Apr 6;11(4):e0152122. doi: 10.1371/journal.pone.0152122 (PMC4822789; doi:10.1371/journal.pone.0152122)
Supplement: S1 Table — In total, 10 skin samples were collected from five Atlantic walruses (three males and two females) in July 2013 (n = 4) and July 2014 (n = 1), as part of the Inuit subsistence hunt in Hudson Strait, near Quaqtaq (Nunavik, Northern Quebec, Canada). Two animals were sampled by LML and three by local collaborators. One skin sample was collected from each of the ventral and dorsal regions of each walrus. The ventral region was designed to act as a negative control, as this region is presumably exposed to little direct UV (other than reflectance). Each sample (cube of 1cm3) included the epidermis and dermis. Upon collection, samples were preserved in the field in 10% buffered formaldehyde solution for later histological analyses. Of the 10 samples collected, one sample (W3NU-S1) was excluded from the analyses, because the sampling location on the body was uncertain. Skin sections were prepared by the Animal Health Laboratory of the University of Guelph. Briefly, the skin fixed in formalin was dehydrated through a series of alcohols, followed by xylene, before being embedded in paraffin wax. The blocks of skin embedded in wax were then sectioned at 4–5 μm and stained with Haematoxylin and Eosin (H&E). Slides were analyzed by LML under 40X magnification, and lesions were semi-quantified following Martinez-Levasseur et al. (2011). Binary response categories (Presence/Absence: 0 = absence and 1 = presence; Levels: 0 = absent or low and 1 = high and widely distributed) were created. NA = not available. (PDF) [file pone.0152122.s002.pdf]

SUPPORTING INFORMATION

File content: The supporting information contains one table entitled: Raw data for the presence and levels of skin abnormalities detected at the microscopic scale in the ventral and dorsal regions of five Atlantic walruses, *Odobenus rosmarus rosmarus*.

Towards a Better Understanding of the Effects of UV on Atlantic Walruses, *Odobenus rosmarus rosmarus*: a Study Combining Histological Data with Local Ecological Knowledge

Laura M. Martinez-Levasseur[1,2,*], Chris M. Furgal[2], Mike O. Hammill[3], Gary Burness[1,*]

[1] Department of Biology, Trent University, Peterborough, Ontario, Canada
[2] Departments of Indigenous Studies and Environmental Resource Studies and Sciences, Trent University, Peterborough, Ontario, Canada
[3] Maurice Lamontagne Institute, Fisheries and Oceans Canada, Mont-Joli, Quebec, Canada

*Corresponding authors
E-mails: garyburness@trentu.ca (GB), lmmartinezlevasseur@gmail.com (LMML)

S1 Table. Raw data for the presence and levels of skin abnormalities detected at the microscopic scale in the ventral and dorsal regions of five Atlantic walruses, *Odobenus rosmarus rosmarus*. In total, 10 skin samples were collected from five Atlantic walruses (three males and two females) in July 2013 (n = 4) and July 2014 (n = 1), as part of the Inuit subsistence hunt in Hudson Strait, near Quaqtaq (Nunavik, Northern Quebec, Canada). Two animals were sampled by LML and three by local collaborators. One skin sample was collected from each of the ventral and dorsal regions of each walrus. The ventral region was designed to act as a negative control, as this region is presumably exposed to little direct UV (other than reflectance). Each sample (cube of $1cm^3$) included the epidermis and dermis. Upon collection, samples were preserved in the field in 10% buffered formaldehyde solution for later histological analyses. Of the 10 samples collected, one sample (W3NU-S1) was excluded from the analyses, because the sampling location on the body was uncertain. Skin sections were prepared by the Animal Health Laboratory of the University of Guelph. Briefly, the skin fixed in formalin was dehydrated through a series of alcohols, followed by xylene, before being embedded in paraffin wax. The blocks of skin embedded in wax were then sectioned at 4 - 5 μm and stained with Haematoxylin and Eosin (H&E). Slides were analyzed by LML under 40X magnification, and lesions were semi-quantified following Martinez-Levasseur et al. (2011). Binary response categories (Presence/Absence: 0 = absence and 1 = presence; Levels: 0 = absent or low and 1=high and widely distributed) were created. NA = not available.

Sample ID	Sampling body location	Sampling year	Walrus sex	Walrus age group	Leukocyte infiltration (absence / presence)	Microvesicles (absence / presence)	Intracellular oedema (low level / high level)	Cytoplasmic vacuolation (low level / high level)	Sunburn cells (low level / high level)	Melanin (absence / presence)	Dendritic melanocytes (absence / presence)
W1NU-S1	back	2013	male	pup	0	0	0	0	1	1	1
W1NU-S2	front	2013	male	pup	0	0	1	0	0	0	1
W2NA-S1	back	2013	female	adult	0	0	0	0	0	1	1
W2NA-S2	front	2013	female	adult	0	0	0	0	0	1	1
W3NU-S1	NA	2013	female	adult	0	0	0	0	0	1	1
W3NU-S2	back	2013	female	adult	0	1	1	0	1	1	1
W4IN-S1	back	2013	male	old	0	1	0	1	0	1	1
W4IN-S2	front	2013	male	old	0	0	0	1	0	1	1
W14-1	front	2014	male	young	0	0	0	0	0	0	0
W14-2	back	2014	male	young	0	1	1	1	0	1	1
